# Psychometric Properties of the Malay Version of the Goal Content for Exercise Questionnaire among Undergraduate Students at the Health Campus, Universiti Sains Malaysia

**DOI:** 10.21315/mjms2019.26.1.11

**Published:** 2019-02-28

**Authors:** Shirlie Chai, Yee Cheng Kueh, Najib Majdi Yaacob, Garry Kuan

**Affiliations:** 1 Unit of Biostatistics and Research Methodology, School of Medical Sciences, Universiti Sains Malaysia, 16150 Kubang Kerian, Kelantan, Malaysia; 2 Exercise and Sports Science, School of Health Sciences, Universiti Sains Malaysia, 16150 Kubang Kerian, Kelantan, Malaysia; 3 Miri Hospital, Jalan Cahaya, 98000, Miri, Sarawak, Malaysia

**Keywords:** self-determination theory, confirmatory factor analysis, construct validity, reliability, goal content, psychometric

## Abstract

**Background:**

Understanding the individual aspirations of exercise participation is important for promoting physical activity. However, there is a lack of evidence to validate a measurement instrument for exercise-based goal content among Malaysian populations. The purpose of this study was to determine the validity and reliability of the Malay version of the Goal Content in Exercise Questionnaire (GCEQ) for a sample of Malaysian undergraduates.

**Methods:**

The original English version of the GCEQ underwent forward and backward translation into the Malay language. A cross-sectional study was conducted. The finalised Malay version was administered to 674 undergraduate students at the Health Campus of the Universiti Sains Malaysia (USM) with a mean age of 20.27 years (SD = 1.35 years). Confirmatory factor analysis (CFA) was conducted for the psychometric evaluation.

**Results:**

The measurement model consisted of 20 observed items and five latent factors. CFA demonstrated adequate fit to the data: comparative fit index = 0.929; standardised root mean square residual = 0.052; root mean square error of approximation = 0.061 (90% CI = 0.056, 0.067). The composite reliability coefficients for the five latent factors ranged from 0.777 to 0.851. All the correlations between the factors were less than 0.85, so discriminant validity was achieved.

**Conclusion:**

The findings suggested that the Malay version of the GCEQ is valid and reliable for assessing goal content in the exercise context of undergraduates at the Health Campus, USM.

## Introduction

Individuals adopt and pursue goals for fulfilment and satisfaction, and different types of goals have manifold consequences. In accordance with Self-Determination Theory (SDT), Sebire et al. (1, 2) developed and tested the Goal Content in Exercise Questionnaire (GCEQ). One of the six mini-theories in SDT, Goal Contents Theory (GCT) is concerned with the goals and aspirations that organise the lives of people. It also has the critical perspective on how these goals and aspirations relate to basic need satisfaction, motivation and wellness.

Goal content is an important predictor of the quality of a person’s behaviour and psychological well-being (2). The pursuit of what is intrinsically meaningful and satisfies basic needs can boost or diminish the wellness and flourish of a person. Consequently, positive outcomes usually are attained with relatively stronger intrinsic than extrinsic aspirations or goals. The contents of a person’s valued goals have an immediate relation to the outcomes, such as well-being. Strong evidence from a longitudinal experiment indicated that intrinsic values group experienced better well-being compared to control group (3).

The World Health Organization (WHO) defined physical activity as ‘any bodily movement produced by skeletal muscles that require energy expenditure’. This definition includes activities performed while working, playing, doing household tasks and engaging in recreational activities. Exercise is a subset of physical activity that is ‘planned, structured, repetitive’ and performed to improve or maintain physical fitness (4). Regular physical activity is a vital element of healthy lifestyle, so insufficient physical activity is viewed as one of the most important risk factors for mortality worldwide. According to the WHO, insufficient physical activity is a contributing factor to the burden of noncommunicable disease, such as cardiovascular diseases, diabetes and cancer (4). It has been reported that more than eight in ten world adolescents and a quarter of adults worldwide are insufficiently physically active (4).

In the Malaysian context, the National Health & Morbidity Survey (NHMS) 2015 carried out by Institute for Public Health reported that the prevalence of physically active adults was 66.5% (5). The inactive Malaysian population was much more prominent compared to the prevalence worldwide (6). According to global statistics in 2010, 23% of adults aged 18 years and older were insufficiently active physically (4). Among all the adults participating in the NHMS 2015, the level of physical activity rose with age, from the 16–19-year-old group to the 40–44-year-old group. Among younger adults, those aged 16–19 years old were the least physically active, merely 61.0% of them were physically active. The group comprised of adults aged 20–24 years was the second least physically active, in which 67.9% of them were physically active (5). It, therefore, is crucial to understand the factors underlying young adults’ motivation to perform physical activity, which is expected to have substantial positive impacts on their future health.

In the sport and exercise psychology setting, SDT is one of the most widely used theories. SDT posits that inherent in intrinsic goal pursuits is satisfaction in one’s competence, autonomy and relatedness (1, 7). Extrinsic goals tend to be less autonomously regulated than intrinsic goals. Compared to extrinsic striving, intrinsic striving has been associated with better outcomes in many samples. In accordance with the theory, Sebire et al. developed the GCEQ to measure intrinsic goal contents (social affiliation, health management and skill development,) and extrinsic goal contents (image and social recognition) in the context of exercise (2). Numerous studies have examined the relationship of aspirations or goals and physical activity participation and have shown that goals have certain impacts on physical activity (8–13). However, studies on the goal content underlying physical activity, particularly among university students who mainly consist of young adults, are lacking (14, 15).

We propose that the goal contents in physical activity participation play a key role in the duration of physical activity among the relatively physically inactive young-adult population. It, therefore, is crucial to understand which goal contents drive and sustain them to undertake physical activity. The Malay version of the GCEQ has importance for future studies on this population whose main language is Malay. This study was aimed at examining the validity and reliability of the Malay version of GCEQ with Malaysian university students.

## Materials and Methods

### Study Design and Procedures

In this cross-sectional study, a questionnaire-based survey using the Malay version of the GCEQ was employed to assess goal content pertaining to exercise. As part of a larger study examining the relationship of several variables, data on exercise were collected using a form to gather demographic details and relevant measures. The study was conducted from December 2017 to April 2018 at the Health Campus, Universiti Sains Malaysia (USM).

### Sample Size Determination

The measurement model was part of a larger study, so the sample size was calculated using the Monte Carlo simulation in Mplus 8 for both the measurement model and the structural relationships of several variables that required larger sample sizes. Based on the simulation results, the minimum sample size to achieve a minimum power of 80% was 500. After considering the 30% non-response rate, the total sample size required was 715 participants. A total of 674 returned the completed questionnaire and were included in the analysis.

### Sampling Method

Purposive sampling, a non-probability-based sampling method, was employed in this study. During recruitment, eligible students were approached after lectures to explain the study and distribute the questionnaire. If the students were interested and willing to participate in the study, they were requested to complete the questionnaire and return it to the researcher. The participants were expected to complete the self-administered questionnaire within 10 min. Participation was voluntary, and the responses had no impact on the students’ academic performance. The participants were reassured that there were no right or wrong answers; they were only requested to answer as truthfully as possible. Implied consent to participate in the study was obtained when they completed and returned the questionnaire.

### Participants

University students at the Health Campus, USM, were recruited as research participants. All the participants were students aged 18 years or older pursuing undergraduate studies on the campus. The participants had strong comprehension of the Malay language and were able to answer the questionnaire. Those available at the time of data collection and willing to spend time answering the questionnaire were invited to participate by completing the questionnaire.

### Measures

#### Demographic/physical and leisure activities information

Several questions captured information on demographics and physical activity. These questions assessed the participants’ characteristics (e.g. age, gender, ethnicity and field of study), the sport or physical and leisure activities in which they were involved and the weekly frequency and duration of their engagement in these activities.

## Goal Content in Exercise Questionnaire

The Malay version of the GCEQ applied. It consists of 20 items and was underpinned by five lower-order factors and two higher-order factors (2). The participants were required to provide responses to all the items rated on a 7-point Likert scale (1 = not at all important; 4 = moderately important; 7 = extremely important). Later, the items were grouped into five subscales: social affiliation, health management, image, social recognition and skill development. Each subscale was measured by four items. The results of a previous study on the original English version of the GCEQ revealed that the five-factor measurement model had excellent model fit (2). The fit indices were: comparative fit index (CFI) = 0.97; standardised root mean square residual (SRMR) = 0.05; root mean square error of approximation (RMSEA) = 0.05 (90% CI = 0.04, 0.06) (2). When the subscales were further categorised as intrinsic and extrinsic goals, the evidence remained convincing: CFI = 0.95; SRMR = 0.07; RMSEA = 0.06 (90% CI = 0.05, 0.07) (2).

### Questionnaire Translation

The original English language version of GCEQ was translated into Malay through the standard forward-and-backward translation method. The fourth author forward translated the English version into Malay based on the principle of retaining the meaning rather than rendering literal word-for-word translation. A bilingual local Malay competent in both Malay and English back translated the Malay version into English. A panel consisting of three experienced experts in sport sciences, sport psychology and psychometric properties carefully reviewed both the forward and backward translations to provide the Malay version with complete representation in terms of phrasing and content with respect to Malaysian culture. All the panel members were competent users of both languages (4). The final version of the GCEQ was pre-tested with ten undergraduate students to collect their feedback on the wording and questionnaire presentation. The results of the pre-test indicated that no modifications were necessary.

### Data Analysis

Descriptive statistics and confirmatory factor analysis (CFA) were performed using IBM SPSS version 24 and Mplus 8, respectively. Prior to the main data analysis, data screening and cleaning were performed to detect erroneous data entry. The assumption of multivariate normality was checked by the Mardia multivariate skewness and kurtosis test. The *P*-values for both tests were significant (*P* < 0.001), indicating that the multivariate normality assumption was violated. Therefore, the maximum likelihood with robust standard errors (MLR) estimator was used.

Hypothesised models have been commonly examined using two ways of evaluating model fit: Chi-square goodness-of-fit statistic and fit indices (16). However, there is no consensus on the appropriate indices for overall goodness-of-fit assessment, so using various types of indices is recommended (17, 18). In the current study, the following goodness-of-fit-indices were used: SRMR, RMSEA with a confidence interval (CI) of 90%, CFI and Tucker-Lewis index (TLI). The sample size and the complexity of the model were considered when determining the cut-off values, as suggested by Hair et al.: SRMR ≤ 0.08 (with CFI > 0.92), RMSEA ≤ 0.07 (with CFI ≥ 0.92), CFI and TLI > 0.92 (18).

Construct validity in CFA consists of two components: convergent and discriminant validity. Convergent validity refers to the extent to which items in the same construct converge or share a high proportion of variance in common. In contrast, discriminant validity is the extent to which two conceptually similar constructs are different in the degree of their correlations with other constructs and how distinctly their items represent only single construct (18).

For analysis of convergent and discriminant validity, various empirical measures were used in this study. Convergent validity was assessed using three criteria. First, statistically significant path estimates fulfilling a threshold of at least 0.5 were used as the reference to confirm that the indicator variables were strongly related to their theoretical construct (18). Composite reliability (CR) was calculated to evaluate the internal consistency of the subscales. We adopted the cut-off value of 0.70 as suggested by the same group of authors (18). The average variance extracted (AVE) was also computed, and a cut-off of 0.5 was adopted (19). Discriminant validity was assessed by evaluating the correlation coefficients between each pair of subscales (20). A value of more than 0.80 or 0.85 for the factor inter-correlation was adopted as an indicator of poor discriminant validity (21).

## Results

### Descriptive Statistics for the Sample

The sample was comprised of 674 undergraduates, 131 male and 543 female, between 18 and 30 years old (mean = 20.27 years, SD = 1.35 years). Their reported ethnicities were Malay (78.3%), Chinese (14.0%), Indian (3.0%) and others (4.6%), while 0.1% did not specify their ethnicity. All the participants were students enrolled in undergraduate studies in Health Campus, USM, in majors such as nutrition, medicine, speech therapy and biomedicine. Of the total sample, 620 self-reported regularly engaging in physical activity at a frequency of 1–7 times per week (mean = 2.77 times, SD = 1.70 times), with a duration of 10–900 min per week (mean = 139.78 min, SD = 139.23 min). The most commonly reported physical activities were jogging, badminton and walking.

### Measurement Model of the Malay Version of the GCEQ

**Figure 1:**
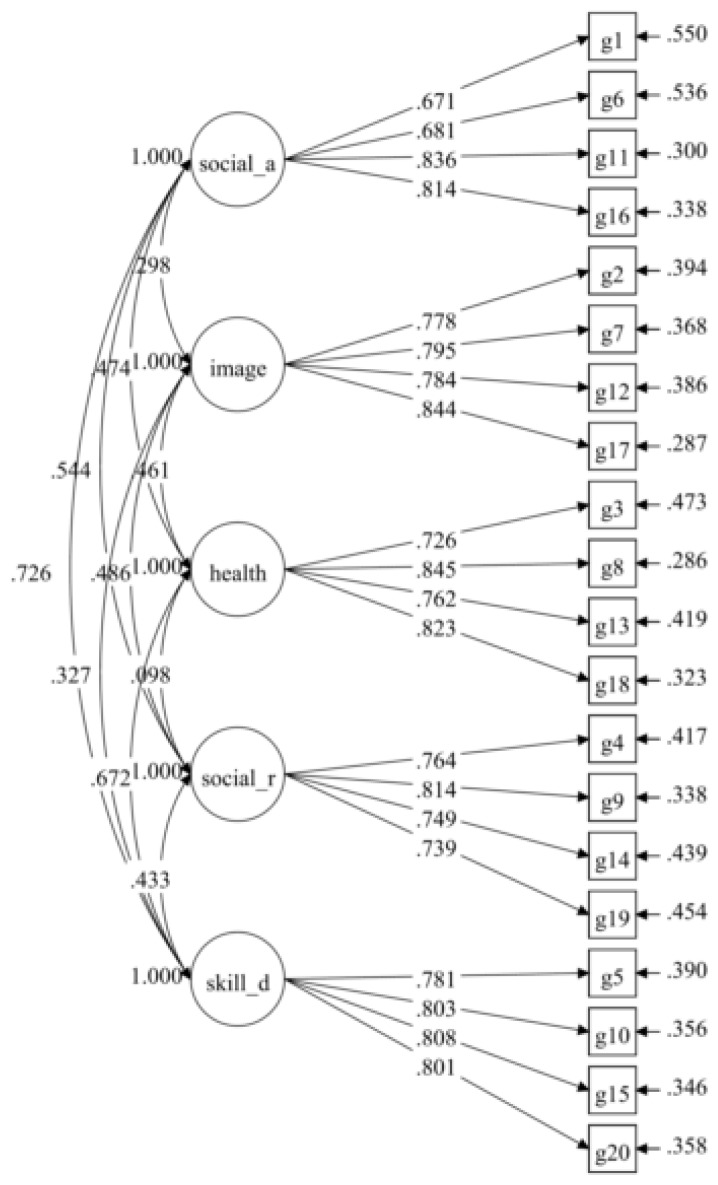
Standardised parameters of the initial model of the Malay version of GCEQ (20 items/five factors)

**Figure 2:**
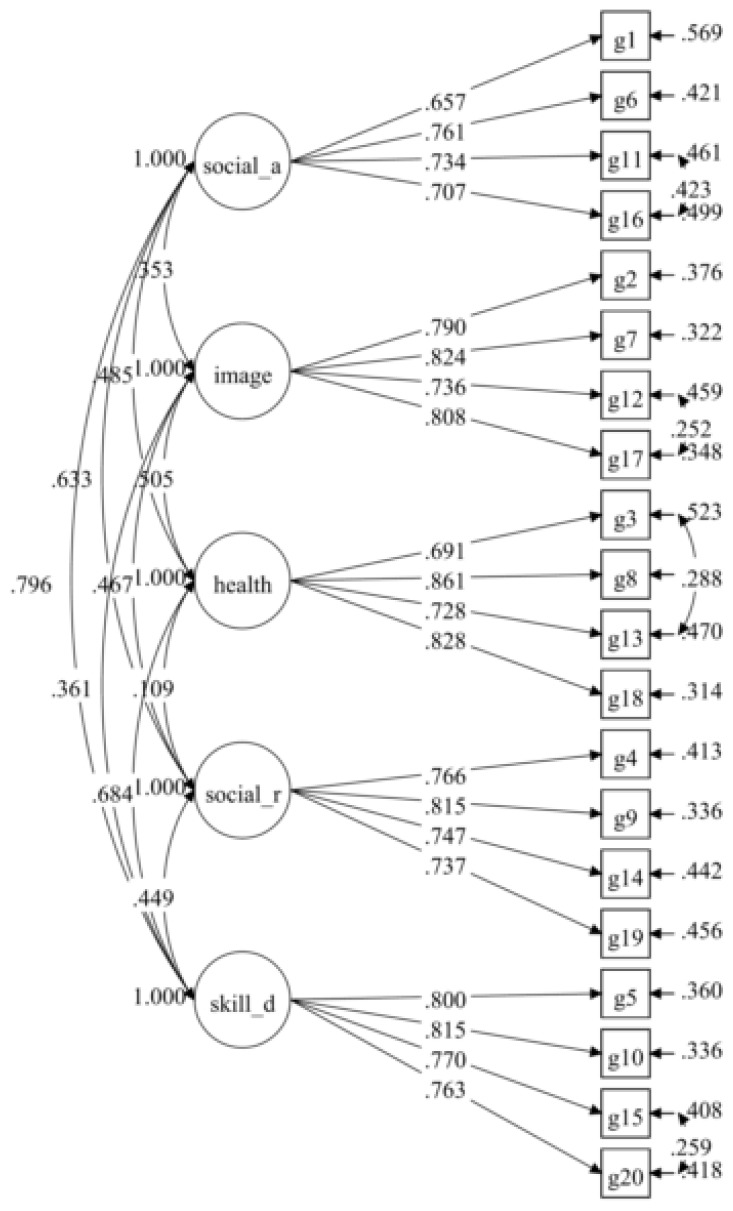
Standardised parameters of the final model of the Malay version of GCEQ (20 items/five factors)

The hypothesised measurement model (initial model) for the Malay version of the GCEQ consisted of five factors with 20 items. The tests of this model did not result in a sufficiently good fit based on several fit indices (Table 1). However, the standardised factor loading for all the items was acceptably good (0.671–0.845) and statistically significant (*P* < 0.001; see Figure 1). The model was improved by correlating of the uniqueness of several items after reviewing the item content, as described in Table 2. The suggested modification to further correlate the errors for items G13 and G18 was not implemented. In the final model, model G(4), the model fit indices were in a desirable pattern, and the standardised factor loadings, ranging from 0.657 to 0.861 were statistically significantly (*P* < 0.05; see Figure 2).

### Convergent and Discriminant Validity

The CR coefficient values were computed for the five subscales in the final model. The reliability values ranged from 0.777 to 0.851. The lowest CR was for Social Affiliation (0.777). Moreover, the AVE values for all the subscales were between 0.512 and 0.624, supporting convergent validity. The factor correlations were all significant but were less than 0.85, demonstrating good discriminant validity. Based on the scale item loadings, CR, AVE and standardised factor covariance, the final model achieved satisfactory convergent and discriminant validity. Table 3 summarises the CR, AVE and standardised factor covariances.

## Discussion

The development of the GCEQ is an important step in assessing exercise goal content (2). One of the major challenges in exercise goal research is measurement of the ‘what’ component of the behaviour. Among the questionnaires measuring the goal contents of physical activity participants, the GCEQ has the most relevant and precise scope. It explicitly addresses the facets of ‘what’ of behaviour, rather than ‘why’ of the behaviour as posited earlier (1). This study was intended to determine the validity and reliability of the Malay version of the GCEQ. The findings showed that the scale was valid and reliable for measuring goal content in the undergraduate sample.

Several fit indices were used to assess the model’s goodness of fit. Researchers have different opinions on the appropriate cut-off values of various fit indices (18, 20, 22–26). The choice of cut-off values remains a controversial but important issue that should be addressed. The arguments, though, are not discussed in this paper. In the current study, we adopted cut-offs that take into consideration the sample size and complexity of the model, as proposed by Hair et al. (18). In the final model, most of the fit indices supported the fitness of the model: CFI = 0.929; TLI = 0.913; SRMR = 0.052; RMSEA = 0.061 (90% CI = 0.056, 0.067). All the fit indices supported good model fit based on the recommended values, except for TLI, which was less than but close to the recommended criteria of 0.92. Based on most fit indices, it is logical and reasonable to accept the final model in this study.

However, several robust estimators in Mplus indicated nonnormality (27, 28). The standard errors and χ^2^ test statistic provided by the MLR estimator are robust to nonnormality (27). Before CFA was performed, the multivariate normality assumption was checked and found to be violated, so the MLR estimator was used. MLR could also be employed to obtain robust estimates when there is missing data and is preferable for small and medium sample sizes (27, 29). Nevertheless, the missing data mechanism which is allowed in the MLR includes missing completely at random and missing at random (27).

The incorporation of the correlated errors within the subscales into the model was performed based on modification index (MI) and justification from a semantical perspective. The item contents were reviewed before the addition of the within-construct error terms. The largest MI (35.934) was noted for items G11 and G16 on Social Affiliation. Both items were reviewed and found to be similar; it, therefore, seemed reasonable to free the covariance between these items’ errors for estimation. A total of four correlated errors was added to form the final model. The justification is shown in Table 2. The fit indices for the final model were improved and satisfactory: CFI = 0.929; TLI = 0.913; SRMR = 0.052; RMSEA = 0.061 (90% CI = 0.056, 0.067). The model fit indices in a desirable pattern were obtained, along with satisfactory standardised factor loadings on the targeted subscale. All the items loaded substantively and significantly on to their respective constructs, so no item was deleted.

From a past study conducted with a Portuguese elderly population, the researchers dropped three items to obtain the final model consisting of five factors with 17 items (30). The model was accepted with the following global fit indices: S-Bχ^2^ = 286.1; *df* = 113; *P* = 0.001; TLI = 0.874; CFI = 0.895; SRMR = 0.077; RMSEA = 0.070 (90% CI = 0.060, 0.080); PCFI = 0.743. Even though the original study had a better fit for the measurement model than the study with Portuguese elderly adults and the present study, we obtained a model with adequate fit and the same number of items as the original scale.

In the current study, the five-factor construct of the GCEQ was appropriate for the undergraduate population. We replicated the findings of Sebire et al. indicating that the goal contents were multidimensional (2). The reliability coefficient values of all the constructs were computed and had a range of 0.777–0.851, indicating good construct reliability. In addition, all the AVE values were more than 0.50. The results showed that the items were strongly associated with their respective factors, supporting convergent validity. In addition to convergent validity, all the correlations between the constructs were less than 0.85, commonly accepted evidence for the distinctiveness of measures. Consequently, discriminant validity was demonstrated, implying that the constructs were sufficiently independent of each other. The multidimensionality of the exercise goal contents thus was evidenced.

Similar to the original study, we tested a second-order model consisting of intrinsic aspirations (social affiliation, health management and skill development) and extrinsic aspirations (image and social recognition). Nonetheless, the measurement model did not show good fit to the data: CFI = 0.885; TLI = 0.866; SRMR = 0.084; RMSEA = 0.076 (90% CI = 0.071, 0.081). Our results were similar to the past study (30). We propose that the interpretations and perceptions of the pursuit of intrinsic and extrinsic goals may be distinct in the sample. The conceptual clarification and differential effects of the intrinsic–extrinsic classification in the younger sample, therefore, may differ from that in the general population in the original study (2).

Consequently, the present study provides new insights into the appropriateness of the five-factor measurement compared to the second-order model (intrinsic versus extrinsic goals) for the undergraduate sample at Health Campus, USM. The research supported the five goal factors representing conceptually and theoretically grounded exercise goals. The five-factor measurement model achieved satisfactory model fit, construct validity and reliability. This finding has practical implications for researchers, health planners and physical educators interested in using the Malay version of the GCEQ to assess individual aspirations for exercise participation. For future studies, we recommend that investigators interpret the goal content scores based on the five-factor model.

The present study has a number of limitations that warrant discussion. First, the study sample was not population based. We included only undergraduate students from one campus of a public university, which may limit generalisability to other populations with different backgrounds, such as private university students and the elderly. It, therefore, is recommended that future work validate these findings in other samples. Second, the present sample with a majority of Malay participants exhibited prominent ethnic homogeneity. In addition, similar to the study by Sebire et al. (2), our sample was largely biased in gender distribution, with more female than male participants.

A further limitation is the cross-sectional nature of the study. Although this is a common study design employed for questionnaire development and validation research, future research could yield valuable information by examining the stability of the GCEQ across time and tests to gain better understanding of the goal pursuits in predicting exercise outcomes, such as the duration of physical activity and the level of enjoyment. Conducting longitudinal studies in this context could provide insightful findings. Longitudinal CFA models could be valuable to examine the stability of the cross-time relationships of the goal contents in the Malay version of the GCEQ and possible changes in the pursuits over time.

## Conclusion

The present study provides validity evidence for the Malay versioned GCEQ. These findings provide new information that translated version of the scale is valid and reliable for goal contents measurement in this undergraduates’ sample. Future research on goal contents for participation in physical activities among the young adults may employ this scale for its aspiration measurement. The interpretation would be appropriate based on the responses for the five-factor framework of subscales.

## Figures and Tables

**Table 1:** Initial and final CFA models of the Malay version of GCEQ

CFA Models	CFI	TLI	SRMR	RMSEA (90% CI)
G(0) Initial	0.911	0.895	0.058	0.067 (0.062, 0.073)
G(4) Final	0.929	0.913	0.052	0.061 (0.056, 0.067)

Notes: CFI = Comparative Fit Index; TLI = Tucker Lewis Index; SRMR = Standard Root Mean Square Residual; RMSEA = Root Mean Square Error of Approximation; CI = Confidence Interval for relevant point estimates

**Table 2:** Global indices of model fit of original and revised models of the Malay version of GCEQ

CFA Models	CFI	TLI	SRMR	RMSEA (90% CI)	Standardised correlation	Justification
G(0) Initial	0.911	0.895	0.058	0.067 (0.062, 0.073)	-	-
G(1)	0.918	0.903	0.054	0.065 (0.059, 0.070)	G11 ~~ G16 (*r* = 0.722)	G11: *Persahabatan yang rapat* (close friendship) G16: *Ikatan rapat* (close bond) Similar statement in Social Affiliation, added the correlated error after reviewing the items content, which indicate similar meaning.
G(2)	0.923	0.908	0.053	0.063 (0.058, 0.069)	G3 ~~ G13 (*r* = 0.908)	G3: *Daya tahan saya terhadap sakit dan penyakit* (resistance to illness and disease) G13: *Keseluruhan kesihatan* (overall health) Both statements in Health Management carry similar meaning, which is referring to one’s health status. Correlated error added.
G(3)	0.927	0.911	0.052	0.062 (0.056, 0.067)	G15 ~~ G20 (*r* = 0.694)	G15: *Menjadi mahir dalam latihan atau aktiviti tertentu* (become skilled at a certain exercise or activity) G20: *Mengembangkan kemahiran senaman saya* (develop my exercise skills) Correlated error added in view of similar statement of items in Skill Development.
G(4) Final	0.929	0.913	0.052	0.061 (0.056, 0.067)	G12 ~~ G17 (*r* = 0.694)	G12: *Menjadi langsing* (to be slim) G17: *Menukar penampilan saya* (to change my appearance) Both items in Image subscale might give similar impression to respondent as slim figure is highly sought after nowadays. Correlated error added.
G(5)	0.930	0.915	0.051	0.061 (0.055, 0.066)	G13 ~~ G18 (*r* = 0.634)	G13: *Keseluruhan kesihatan* (overall health) G18: *Ketahanan dan stamina* (endurance, stamina) Correlated error was not added for the items because they are deemed dissimilar although they are from the same subscale.

Notes: CFI = Comparative Fit Index; TLI = Tucker Lewis Index; SRMR = Standard Root Mean Square Residual; RMSEA = Root Mean Square Error of Approximation; CI = Confidence Interval for relevant point estimates.

**Table 3:** CR, AVE, and standardised covariance between factors of the final model of Malay version of GCEQ

	CR	AVE	1	2	3	4	5
1. Social Affiliation	0.777	0.512	1	0.353*	0.485*	0.633*	0.796*
2. Image	0.850	0.624		1	0.505*	0.467*	0.361*
3. Health Management	0.839	0.609			1	0.109*	0.684*
4. Social Recognition	0.851	0.588				1	0.449*
5. Skill Development	0.850	0.620					1

* Standardised covariance is significant at the 0.05 level (two-tailed),

CR = composite reliability, AVE = average variance extracted
